# Supplementary material for: Photon upconversion with directed emission
Source: Nat Commun. 2016 Aug 30;7:12689. doi: 10.1038/ncomms12689 (PMC5013605; doi:10.1038/ncomms12689)
Supplement: Supplementary Information — Supplementary Figures 1-7 and Supplementary Note 1 [file ncomms12689-s1.pdf]

Supplementary Figures

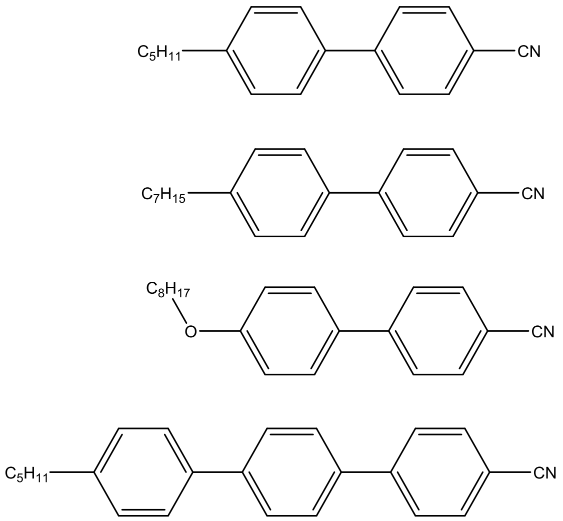

5CB, 51%

7CB, 25%

8OCB, 16%

5CT, 8%

Supplementary Figure 1 The liquid crystalline matrix (E7) used comprising of from top to bottom (weight% of each component in parenthesis): 4-cyano-4'-n-pentyl-1,1'-biphenyl (51%), 4-cyano-4'-n-heptyl-1,1'-biphenyl (25%), 4-cyano-4'-n-octyloxy-1,1'-biphenyl (16%), and 4-cyano-4''-n-pentyl-1,1',1''-terphenyl (8%).

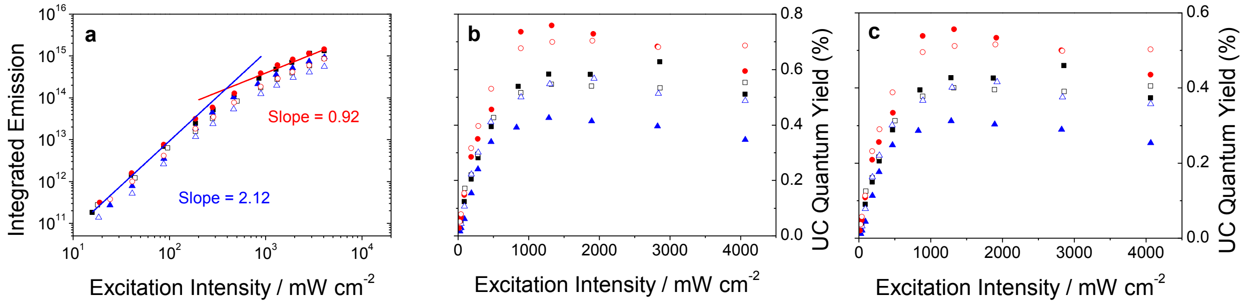

Supplementary Figure 2 Upconverted emission as a function of excitation intensity (**a**). Red and blue solid lines indicate linear and quadratic regions, respectively. Uponversion quantum yields as a function of excitation intensity using 1.54 (**b**) and 1.8 (**c**) as refractive indexes for E7, respectively (the refractive index of E7 is 1.80 and 1.54 for light parallel and perpendicular to the matrix directors respectively, therefor the quantum yield is reported in the interval of the two extremes). The concentration of annihilator was 1 mM in all cases (**1**: red circles; **2** black squares; and **3** blue triangles), and the concentration of **4** was 100 μM (filled) or 50 μM (open).

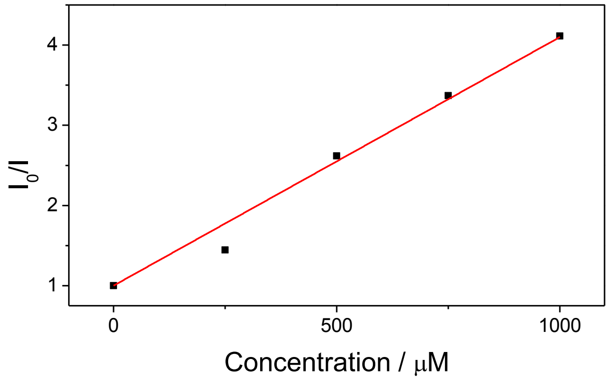

Supplementary Figure 3 Stern-Volmer plot showing the quenching of **4** phosphorescence as a function of concentration of **1**. The linear regression gives a Stern-Volmer quenching constant of 3100 M^{-1}, which is translated to a bimolecular quenching constant of $1.55 \cdot 10^{7}$ M^{-1} s^{-1} using the natural lifetime of **4**.

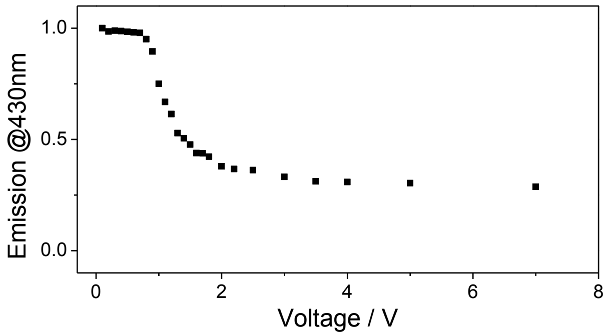

Supplementary Figure 4 Upconverted emission as a function of applied AC-voltage. Concentration of **1** and **4** was 1 mM and 100 μM, respectively, and excitation wavelength was 547 nm.

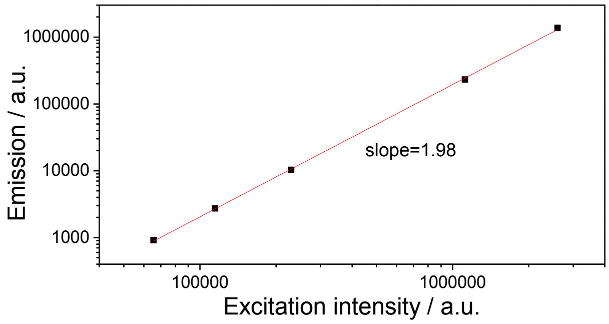

Supplementary Figure 5 Upconverted emission as a function of excitation intensity showing a quadratic dependence. Concentration of **2** and **4** was 1 mM and 100 μM, respectively, and excitation wavelength was 547 nm.

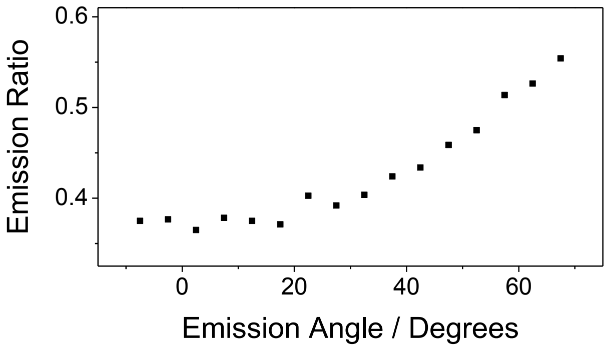

Supplementary Figure 6 Ratio of upconverted emission as function of emission angle (emission orthogonal to the device surface is at 0°). Excitation wavelength was 547 nm and the emission was monitored at 430 nm. The emission angle was changed by rotating the device in the sample chamber of the spectrophotometer. To correct for differences in excitation light intensity, the emission in presence of an applied field was divided by the emission without applied field, giving an emission ratio. The alignment layer is positioned in a vertical position, which means that in the absence of reflections at sample interfaces the theoretical ratio of emission, should vary between 0.37 at 0 degrees and 1 at 90 degrees.

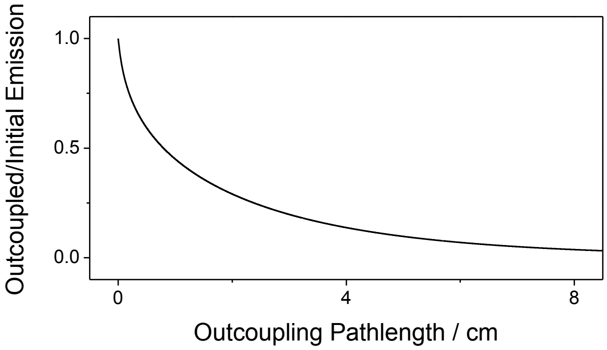

Supplementary Figure 7 Simulation of self-absorption losses of the upconverted light as a function of distance travelled through the upconversion media by the out coupled light. In this simulation the effect of reabsorption by the sensitizer is calculated, showing that reabsorption by the sensitizer of the out coupled light, at the experimental conditions used, is an issue at the cm length scale.

Supplementary Notes

Supplementary Note 1: Analysis of fraction oriented molecules and system efficiency The assessment of system efficiency using **1** as emitter was done using the data presented in Fig. 5. It is assumed that the emission mathematically can be explained by one perfectly and one randomly oriented population. All emission from the cases with an applied electric field as well as with alignment layer and polarizer in orthogonal positions purely results from the randomly oriented population. For the two cases with parallel alignment layer and polarizer the emission comes both from the perfectly and randomly oriented populations. The total emission (t) for samples with alignment layer and polarizers parallel can be expressed as a combination of emission from random (r) and oriented (o) populations.

$$I_{\mathrm{r}} + I_{\mathrm{o}} = I_{\mathrm{t}}$$ Supplementary Equation 1

The average emission strength ratio for the six cases (see Fig. 5) with purely randomly (r) oriented samples can be expressed by:

$$\frac{I_{\mathrm{r}}}{I_{\mathrm{t}}} = \frac{(I_{\mathrm{H,V,0V}} + I_{\mathrm{H,V,14V}} + I_{\mathrm{H,H,14V}} + I_{\mathrm{V,H,0V}} + I_{\mathrm{V,H,14V}} + I_{\mathrm{V,V,14V}})}{6} = 0.316$$ Supplementary Equation 2

Where the indexes ($I_{\mathrm{x,y,z}}$) indicate macroscopic alignment, polarizer direction, and applied voltage, respectively. The emission intensity of a population depends on the mole fraction (X) of the population and the emission efficiency (E) of the population.

$$I = X \cdot E$$ Supplementary Equation 3

The emission efficiency in the direction of the oriented population is twice as strong as that for the randomly oriented population.

$$E_{\mathrm{o}} = 2 \cdot E_{\mathrm{r}}$$ Supplementary Equation 4

Combining equations 1 and 3 gives:

$$X_{\mathrm{o}} \cdot E_{\mathrm{o}} = I_{\mathrm{o}} = I_{\mathrm{t}} - I_{\mathrm{r}}$$ Supplementary Equation 5

$$E_{\mathrm{o}} = \frac{I_{\mathrm{t}} - I_{\mathrm{r}}}{X_{\mathrm{o}}}$$ Supplementary Equation 6

And combining equation 3 and 4 gives:

$$X_{\mathrm{r}} \cdot E_{\mathrm{r}} = (1 - X_{\mathrm{o}}) \cdot 0.5E_{\mathrm{o}} = I_{\mathrm{r}}$$ Supplementary Equation 7

$$0.5E_{\mathrm{o}} - 0.5E_{\mathrm{o}}X_{\mathrm{o}} = I_{\mathrm{r}}$$ Supplementary Equation 8

Substitution of equation 6 into 8 results in:

$$\frac{I_{\mathrm{t}} - I_{\mathrm{r}}}{X_{\mathrm{o}}} - I_{\mathrm{t}} + I_{\mathrm{r}} = 2I_{\mathrm{r}}$$ Supplementary Equation 9

$$I_{\mathrm{t}} - I_{\mathrm{r}} - I_{\mathrm{t}}X_{\mathrm{o}} + I_{\mathrm{r}}X_{\mathrm{o}} = 2I_{\mathrm{r}}X_{\mathrm{o}}$$ Supplementary Equation 10

$$I_\mathrm{t} - I_\mathrm{r} = I_\mathrm{r} X_\mathrm{o} + I_\mathrm{t} X_\mathrm{o} = (I_\mathrm{r} + I_\mathrm{t}) X_\mathrm{o}$$ Supplementary Equation 11

$$X_\mathrm{o} = \frac{I_\mathrm{t} - I_\mathrm{r}}{I_\mathrm{r} + I_\mathrm{t}} = 0.52$$ Supplementary Equation 12

Having the mole fraction of perfectly oriented molecules, the directivity of the system can be calculated by comparing the emission at zero degrees compared to the average emission.

$$\alpha = \frac{\sum X \cdot E}{E_\mathrm{r}} = \frac{X_\mathrm{o} \cdot E_\mathrm{o} + X_\mathrm{r} \cdot E_\mathrm{r}}{E_\mathrm{r}} = \frac{X_\mathrm{o} \cdot 2E_\mathrm{r} + X_\mathrm{r} \cdot E_\mathrm{r}}{E_\mathrm{r}} = 2X_\mathrm{o} + X_\mathrm{r} = 1.52$$ Supplementary Equation 13
